# Postmortem sperm retrieval for *in vitro* fertilization treatment: care to be taken - a Brazilian case report

**DOI:** 10.5935/1518-0557.20140013

**Published:** 2014

**Authors:** Edilberto Araújo Filho, Cássio L. Fácio, Luís Antonio Velani, Rui N. Barbosa, Ligiane A. Machado-Paula, Lígia F. Previato

**Affiliations:** 1 Center of Human Reproduction of Sao Jose do Rio Preto, São José do Rio Preto - SP, Brazil

**Keywords:** FSH, Nr4A1, Egr1, Gene transcription, GVBD

## Abstract

Postmortem sperm retrieval has been used worldwide in assisted reproduction technology. Nevertheless, the laws vary from country to country according to cultural, ethical and religious reasons. However, for postmortem sperm retrieval to be used, it is necessary that a preview informed consent be signed by the couple. In this study, we describe a case where the husband died during the *in vitro* fertilization (IVF) treatment prior to egg retrieval, but we had no informed consent with a paragraph concerning this issue. Thus, the wife had to request a judicial authorization, which was given in the case of an emergency by the judge, to retrieve genetic material from her husband after his sudden death. This case report demonstrates the importance of adding a specific paragraph in the informed consent to address this issue. Otherwise, this case may be a cause of a judicial battle to obtain consent for its use in *in vitro* fertilization.

## INTRODUCTION

Postmortem sperm retrieval (PMSR) is the method of obtaining semen from a recently deceased male to be used in assisted reproduction technology (ART) at a later time (Ahuja *et al*., 1997). The first PMSR was reported in 1980 by Rothman in a case involving a 30-year old man with brain death after a motor vehicle accident (Rothman, 1980). In the USA between 1980 and July 1995, a total of 40 centers reported 82 requests for PMRS (Webb, 1996; Kerr *et al*., 1997), and from 1997 to 2002, the number of requests increased by 60% and the number of retrievals by 68% (Hurwitz & Batzer, 2004). Despite these increased requests, there are currently no governing guidelines and protocols in the USA thus, it is difficult to make fast and efficient decisions regarding PMRS (Smith & Lipshultz, 2013).

PMSR and its utilization in ART are performed worldwide. However there were divergences due to differences in national culture, ethical values and legislation (Benshushan & Schenker, 1998; Dostal *et al*., 2005). There are no laws and regulations for this type of intervention in all countries, and they may also differ from country to country (Epker *et al*., 2012).

Prior informed consent of the male should be considered as a basic prerequisite for sperm retrieval because an unexpected death can occur, and if an informed consent is not available, then the result will be a complex legal situation to solve for the surviving partner or the parents of the deceased or even another person who may request the PMSR (R. v HFEA, 1997). According to Dostal *et al*. (2005) in this type of situation, which demands rapid action, the multidisciplinary team involved is confronted with a number of difficult ethical and legal concerns.

Guidelines for PMSR in the medical literature suggest that the procedure should be performed within 24 h after death to obtain motile or vital sperm (Webb, 1996; Land & Ross, 2002; Tash *et al*., 2003). According to Shefi *et al*. (2006), viable sperm can be obtained with PMSR even after the currently recommended 24 h time interval, and viable pregnancies are also possible with sperm retrieved after this limit. Moreover, PMSR should be considered up to 36 h after death following an appropriate evaluation.

The objective of this case report was to inform *in vitro* fertilization (IVF) centers concerning the possibility of finding viable sperms in PMSR, and to encourage an insertion of a paragraph in the informed consent on PMSR such that the couples choose, before the treatment, what they want to do. This information will help IVF centers and families to determine how to proceed if such a situation occurs.

## CASE DESCRIPTION

### Patients

A 58-year-old male and 31-year-old female began an IVF treatment at the Center of Human Reproduction of Sao Jose do Rio Preto, SP. They tried IVF treatment twice in another reproductive center. She became pregnant once but experienced a miscarriage in the first trimester. The couple signed an informed consent prior to the start of treatment. However, these informed consent did not have not a paragraph concerning PMRS. Today, we included this paragraph as the following: “We declare that after signing this document, from the beginning of the treatment, in case of death of either partner, we wish that our gametes (oocytes and sperm) are available to the surviving spouse, who will have the autonomy to decide about their destination.”

The patient reported menarche at 12 years of age, regular menstrual cycles, three days of bleeding, and no cramps or dyspareunia. The test results for thyroid and prolactin were normal, and transvaginal ultrasound examination did not show pathologies.

The husband had severe oligospermia due to mumps orchitis. He had two children by natural pregnancy from a previous marriage before the orchitis. Moreover, he was carrier of Chagas disease with controlled cardiac arrhythmia.

The spermogram tests results were the following: volume 1.0 ml; concentration 3 million/ml; motility 33% (Grade 2: 16%; Grade 3: 17%); normal morphology 17% (WHO, 2010).

Sperm organelle morphology examination (MSOME) was performed: the presence of vacuoles 79% (small vacuoles 35%; large vacuoles 28%; multiple vacuoles 16%); absence of vacuoles 21% (no vacuoles but with defect in the midpiece and/or tail 19%; no vacuoles and no defects 2%) (Bartoov *et al*., 2002).

## Methodology

### Ovarian stimulation protocol

The patient received oral contraceptive on the cycle before ovarian stimulation. She received 225 IU of recombinant follicle stimulating hormone (r-FSH; Gonal F, Serono, Brazil) on days 2 to 4 of the menstrual cycle, 150 IU on days 5 and 6 of the cycle and a vaginal ultrasound on day 7. The gonadotrophin-releasing hormone (GnRH) antagonist (Cetrotide, Serono, Brazil) was initiated when the follicular diameter reached 16 mm in diameter, which occurred on day 8 of the menstrual cycle and was continued until the day before the oocyte retrieval. From day 8 onward, an ultrasound was performed daily. When two to three follicles measured 20mm, 250mcg recombinant human chorionic gonadotropin (r-hCG; Ovidrel, Serono, Brazil) was administered as an ovulation trigger.

On day 9 of the menstrual cycle (October 2011), during the ultrasound, the husband did not feel well in the waiting room and fainted while having a cardiopulmonary arrest. Cardiopulmonary resuscitation was performed immediately, and the patient was taken to the hospital, where he died two hours later for non-reversible ventricular fibrillation.

His wife inquired into the possibility of PMSR. When we consulted our lawyer regarding the legality of this procedure, he informed us that our informed consent had no paragraph concerning PMSR. Under this circumstance, our lawyer decided to request, in case of an emergency, an “injunction of authorization for testicular sperm aspiration post-mortem,” to the judge in charge that day, and he promptly authorized the procedure, but the freezing material would not be able to be used for IVF at that time.

The judge also referred that the applicant had to obtain judicial authorization to use the frozen sperm because the informed consent had no mention of such an event.

Five hours after the husband’s death, the urologist and chief embryologist of our clinic performed the epididymal sperm aspiration bilaterally and obtained the following seminal sample: volume 2.0 ml; total concentration 1,000,000 sperm; motility 30% (Grades 3 and 4) and normal morphology 15% (WHO, 2010). The semen was frozen after analysis. For this procedure, the semen sample (volume 2.0 ml) was placed into a 15 ml conical Falcon tube, and an equal volume of 2.0 ml of cryoprotector media was added (Freezing Medium, Irvine Scientific) (proportion 1:1), which had been previously acclimated to room temperature. The final 4 ml volume was aliquoted into two cryotubes (Corning Incorporated); each cryo-tube was capable of holding 2.0 ml of mixture. These cryotubes were stored for 20 minutes at 2-8 degree Celsius followed by10 minutes in liquid nitrogen vapor (N2L). Next, the cryotubes were placed in a metal rack and stored in a liquid nitrogen container at a previously determined location and identified. The wife underwent transvaginal ultrasound-guided oocyte retrieval when the follicles reached 20 mm in diameter four days after the husband’s death. She had 8 follicles on the right ovary and 8 follicles on the left ovary. Fifteen mature oocytes were retrieved and vitrified.

## DISCUSSION

It is known that there is a lack of consensus with regards to the use of PMSR on the national, international and institutional levels (Smith & Lipshultz, 2013).

According to Batzer *et al*. (2003), a number of countries have a formal regulatory policy, while others do not, and as the number of requests for these procedures increases, we have to prepare ourselves ethically and legally for this situation. Bahm *et al*. (2013) analyzed existing protocols of PMRS in the USA and provided six important considerations to help institutions to develop an institutional policy regarding this issue with practice guidelines protecting institutional and individual liability.

Modern ARTs have reinforced the feasibility of PMSR but have also brought to light many moral, ethical, legal and social issues (Bahadur, 2002; Land & Ross, 2002; Orr & Siegler, 2002; Batzer *et al*., 2003; Pastuszak *et al*., 2013). There are a several guidelines on PMSR in North American and European jurisdictions, but in most of these guidelines, none of the regulations is in place. In 2004, the guidelines of the American Society of Reproductive Medicine (ASRM) suggested that PMSR should be offered only if the deceased had given prior consent or if his wish to retrieve sperm was widely known (Ethics Committee of the American Society for Reproductive Medicine, 2004). For the European Society of Human Reproduction and Endocrinology (ESHRE), the guidelines provided in 2006 recommended that PMSR should be offered only if the deceased had provided written consent, and the sperm should be used only after extensive counseling and at least one year after the death (Pennings *et al.,* 2006).

Thus, there is a general consensus in several guidelines to not use the semen for a period from six to 12 months, with appropriate medical and psychological evaluation being performed beforehand (Gottinger & Nagler, 1999; Soules, 1999; Rubinstein, 2003), because bereavement can strongly affect important decisions (Raziel *et al*., 2011).

In Canada, PMSR is not allowed without a written informed consent from the deceased, and even with a written consent, certain ethical and social issues regarding this procedure should be discussed with the family, such as the costs of the fertility procedures and costs involved in raising the child, the social stigma of raising a child without a biological father and estate distribution (Weber *et al*., 2009).

In Brazil, in 2010, a postmortem ART was added to the Federal Council of Medicine guidelines, which enabled this procedure to be performed after the death of either parent if there were a prior written consent that permitted the cryopreservation of gametes or embryos.

This case report is important to alert the IVF centers to the possibility of obtaining viable sperm after death. According to Shefi *et al*. (2006), viable sperm can be obtained using PMSR even after the currently recommended 24h time interval, and viable pregnancies are possible when sperm is retrieved after this period. Moreover, PMSR should be considered for up to 36h after death following an appropriate evaluation.

Another important point is the inclusion of a specific paragraph in the informed consent concerning authorization for retrieval of the gamete (sperm or oocytes) of either partner in the case of death during treatment such that the surviving partner can continue the treatment to the end. This treatment was not possible in the present case and is already a target of a judicial dispute regarding whether to use the material obtained from the husband. The sperm sample is still frozen. To date, the widow has not obtained judicial authorization to use the frozen sperm.
